# Stories of hope created together: A pilot, school-based workshop for sharing eco-emotions and creating an actively hopeful vision of the future

**DOI:** 10.3389/fpsyg.2022.1076322

**Published:** 2023-01-06

**Authors:** Elizabeth Marks, Ed Atkins, Joanne K. Garrett, Jesse F. Abrams, David Shackleton, Lauren Hennessy, Elouise E. Mayall, James Bennett, Isabel Leach

**Affiliations:** ^1^Department of Psychology, University of Bath, Bath, United Kingdom; ^2^School of Geographical Sciences, University of Bristol, Bristol, United Kingdom; ^3^European Centre for Environment and Human Health, University of Exeter, Truro, United Kingdom; ^4^Global Systems Institute, University of Exeter, Exeter, United Kingdom; ^5^School of English, Communication and Philosophy, Cardiff University, Cardiff, United Kingdom; ^6^Climate Change Education Research Network (CCERN), Bristol, United Kingdom; ^7^University of Bristol, Bristol, United Kingdom; ^8^School of Biological Sciences, University of Aberdeen, Aberdeen, United Kingdom; ^9^Biological and Environmental Sciences, Faculty of Natural Sciences, University of Stirling, Stirling, United Kingdom; ^10^School of Earth and Environment, University of Leeds, Leeds, United Kingdom

**Keywords:** eco-emotions, climate anxiety, hope, young people, schools, story-telling

## Abstract

The climate and ecological crises challenge all communities across the world, with the greatest impact upon the most vulnerable and the youngest. There are multiple impacts on mental health, including the psychological burdens that arise with increasing awareness of the loss, threat and injustice caused by these crises. Large numbers of young people globally are understandably concerned and distressed about these crises, whilst simultaneously reporting that their concerns are regularly dismissed and ignored, particularly by those in power. This can increase feelings of isolation and distress, particularly if they have no recourse to effect change. This pilot project sought to explore how a schools-based, co-created workshop for school pupils aged 16 to 18 years could use a community-oriented space to explore their eco-emotions, address feelings of isolation and engender a sense of realistic, active hope, using storytelling and images of possible futures. A 3-h workshop for delivery in schools was co-designed with young people, researchers, educators and clinicians, using principles of Youth Participatory Action Research (YPAR). Six school pupils aged 16–18 years consented and four completed the workshop, which involved a range of group-based activities to explore their understanding of the climate and ecological crises, support emotional expression related to these and engage in storytelling about hopeful and realistic futures. A live illustrator in attendance created shared images of the participants’ fears and hopes. The workshop was recorded, transcribed and analysed using Thematic Analysis and sentiment analysis. Feedback was sought from participants at 1 and 4 weeks after completion and analysed using content analysis. Results indicated that participants reported a range of painful and positive emotions about the crises. They highly valued having space to express their experience alongside others. Storytelling and creativity appeared to help them articulate their feelings and hopes for the future, and gave them greater motivation and confidence in talking to others about these topics. This innovative pilot study suggests that a school-based youth participatory group could offer a novel way of helping young people to engage more with the climate and ecological crises in a way that supports their wellbeing. It provides strong support for future, larger-scale projects in this area.

## Introduction

Climate change represents an existential crisis for humanity, and securing a liveable future for humankind requires immediate action to mitigate and adapt to the ongoing climate crisis (IPCC, 2022). Climate change and ecological breakdown are interlinked challenges threatening all nations. The most vulnerable and marginalised global communities, children and future generations will suffer the most, with an estimated 1 billion children living in the 33 countries most likely to experience multiple climate shocks (UNICEF, 2021). Climate change will affect mental health through multiple pathways (Berry et al., 2018). This includes proximate impacts (e.g. exposure to extreme weather events), intermediate impacts (e.g. through adverse changes to socio-cultural structures due to climate change) and distal impacts (e.g. through distress arising from awareness of the crises).

The reality of the significant threat to the health and wellbeing of humans and other species means that painful cognitive-emotional responses are understandable and rational (Pihkala, 2020). Such responses have been termed ‘climate anxiety’, ‘eco-anxiety’, ‘climate grief’, ‘solastalgia’ or ‘eco-distress’, and younger people are particularly likely to report such worries (Vergunst and Berry, 2021). A recent global study of 10,000 people aged 16–25 years indicated that 60% feel very or extremely worried about climate change, with 45% reporting that such concerns affect their daily functioning (Hickman et al., 2021). Emotions linked to climate change included fear, sadness, anxiety, powerlessness, helplessness, guilt, depression, despair and grief. Young people here reported negative thoughts, with 75% thinking that ‘the future is frightening’ and 56% believing that ‘humanity is doomed’. These painful thoughts and feelings were associated with feeling betrayed by government inaction on climate change. Due to their developmental stage, young people are vulnerable to the effects of multiple stressors and long-term impact of disease and chronic stress. They also have less agency to mitigate or adapt to such stressors. Evidence for widespread climate distress in young people suggests how awareness of climate change represents a chronic psychological stressor which could negatively impact their mental health and wellbeing (Hickman et al., 2021; Vergunst and Berry, 2021).

Despite this concern, many young people feel ignored or dismissed when attempting to discuss such concerns and feelings (Hickman et al., 2021). This attempt to protect them, so as not to ‘frighten the children’ (Hickman, 2020) in fact makes them feel unsupported and marginalised (Baker et al., 2021). Such attempts cannot prevent young people accessing plentiful information about climate change *via* mainstream and social media (Rousell and Cutter-Mackenzie-Knowles, 2019), although again their perspectives are very rarely represented in mainstream press (Graham and de Bell, 2020). School responses so far tend to include some climate change education, but this is largely a science-based, top-down approach, with little reference to emotional, psychological, sociopolitical or ethical factors. As such, young people are fully aware of the crises and are emotionally affected by them, but adults are not empowering them to respond or supporting them with the psychological burdens (Rousell and Cutter-Mackenzie-Knowles, 2019).

A new approach is needed, particularly as evidence indicates that sharing eco-emotions and collective engagement can reduce climate distress and despair in young people and engender hope (Ojala, 2007; Hickman, 2020). This chimes with research into hope in healthcare settings (Olsman, 2020), which indicates that inspiring relationships with peers who share your experiences increase hope. This is because hope requires an awareness of, and openness to possible future outcomes, an ability to respond to this creatively, resilience (an ability to endure adversity) and an opportunity to identify meaning and values and act in line with these. This requires a balance between speaking the truth about a threat, expressing emotion and values and finding a way to keep the door open for hope (Olsman, 2020, p.202).

Balancing truth and hope in eco-emotions requires engagement with action (‘active’ or ‘constructive’ hope; Ojala, 2012). This is different from denial, disavowal, avoidance or apathy, because active hope demands that we hold two competing realisations in mind: (1) the reality of the crises *and* (2) that change and a sustainable future is possible, *if* we act together now. Realistic hope can be held alongside the more painful aspects of eco-anxiety, supporting resilience. Hope is essential in enabling individuals to recognise both their capabilities and the opportunities available to them in a way that can help them engage in behavioural change (e.g. COM-B model; Michie et al., 2014). This theory is supported by findings that higher levels of eco-emotions is linked to more climate action and constructive, pro-environmental behaviours, and that this is also associated with environmental identity (Helm et al., 2018; Verplanken et al., 2020; Whitmarsh et al., 2022).

Eco-emotions are a sign of care for people, planet and other species, a care that motivates behavioural change, and in this way are also a sign of eco-empathy and eco-compassion, related concepts which arise from the motivational system of caring for others (Gilbert, 2015). It may be worth differentiating between these two states, based on psychological understanding of the differences between empathy and compassion. A helpful distinction can regard empathy as an affective mode of understanding, which leads to feel what the other is feeling. Empathy can translate into ‘empathic concern’ if it focuses on distress and motivates care. Compassion similarly involves recognising suffering (empathic awareness), and tends to involve an affective element, where one is moved by such suffering, combined with both a wish for the suffering to be alleviated, and motivation to act in a way to help (see Gilbert, 2015 for further discussion). In the case of eco-empathy and eco-compassion, we can see these are responses that arise as people become aware of the suffering arising with the climate and ecological emergency, affecting many other people, species, eco-systems and even the whole planet. Eco-empathy about the crises would thus involve feeling moved and distressed by recognition of associated suffering. Eco-compassion would involve a similar affective element, but also include motivation to reduce the suffering through pro-social action and care, and possibly involve more-positive emotions as a result (Singer and Klimecki, 2014).

The Department for Education’s (2022) strategy on sustainability and climate change acknowledges the worries faced by young people: ‘Children and young people are worried about climate change and want to know more about: the impact it is having now; how it will impact their future lives’ (from DfE strategy, 2022). Despite this acknowledgement, the strategy provides very little reference to the emotional experience of young people or the potential role of schools in providing a space for emotions. Given that young people will be exposed to distressing facts about the scale of the ecological crisis, there is a responsibility on educators to facilitate safe spaces for young people’s emotional realities to be acknowledged. Currently, teachers are not trained to do this, and schools are not set up to support this work. It is vital to find new ways of resourcing schools to support active hope and emotional resilience in the face of climate distress. The importance of finding new strategies to help with this was elucidated by Pihkala (2020) who indicates the importance of providing spaces where ecological emotions can be discussed, validated and engaged with in a way that offers ‘embodied and creative activities to more fully deliberate on them’.

Storytelling can be a powerful tool for helping people to engage emotionally with critical situations. Emerging evidence shows the benefits of storytelling workshops to help work constructively with overly negative public discourses about climate change (Harcourt et al., 2021). Telling stories about one’s life and experiences is a powerful way to shift perception of oneself as it creates an opportunity to see life within a broader perspective, including sociopolitical and cultural contexts. Narrative therapies are based on this idea, and by inviting people to take a view of their life, they are able to separate what has often become an internalised difficulty which can feel merged with the self (‘externalising’ a problem). This creates space to tell new stories of preferred lives (White and Epston, 1990), which the individual can then work towards. This can be grounded in the narrative identity theory of Dan P. McAdams, which asserts that identity itself is developed, and continues to evolve, as people internalise a life story, where they integrate past experiences and imagined futures, and that this is an important process to give a sense of meaning and purpose to their lives (McAdams, 2001). Culturally, this is particularly pertinent for adolescents, a life stage characterised by identity formation process (Kroger et al., 2010).

There is also clear evidence that research should use co-creation methodologies that recognise and harness the expertise held by participants and stakeholders by involving them fully throughout the research process. Co-creation depends upon developing trusting, collaborative relationships between participants and researchers and addressing hierarchical barriers (Brown, 2022) and power dynamics (Smyth and Williamson, 2004). As eco-distress is a potentially sensitive and painful topic (Pihkala, 2020), linked to a lack of trust in authority (Hickman et al., 2021), and lacking in empirical evidence, any research in this area must redress such imbalance and amplify young people’s voices whilst offering a safe, supportive and empowering space (e.g. Hart, 1992). Youth Participatory Action Research (YPAR) is particularly supportive of working with adolescents on areas that support health and wellbeing, and is a way for adults to learn from, and with young people in a way that can empower them (Ozer, 2017). PAR is based on an epistemological foundation that all individuals are experts, and that research should integrate the knowledge of those being ‘researched’ within the research process (Caraballo et al., 2017). Principles of YPAR aim to engage youth in systematic research procedures alongside adult researchers, and focuses particularly upon social injustice and transformation (Cammarota and Fine, 2008b), making it particularly relevant to ecological issues.

### Aims and objectives

This pilot project aimed to find new ways of understanding and responding to eco-emotions and exploring ways of developing hope in school pupils (aged 16–18 years). A workshop was designed through co-production with youth collaborators (aged 19-25), following principles of YPAR, such as power sharing and youth-led decision-making. This youth-led design process produced a workshop plan that offered a psychologically safe space for young people to come together to express and explore their knowledge, thoughts and feelings about climate change through storytelling, visual imagery and discussion of hoped-for futures.

### Research questions

1. How do young people share their thoughts and feelings about climate change in a peer-group setting?
2. In what ways do these thoughts and feelings take the form of stories and/or recognised eco-emotions?
3. In what ways might peer-group discussions be co-created to bring about stories of hope in climate change, alongside more painful eco-emotions?

## Materials and methods

**Figure 1:**
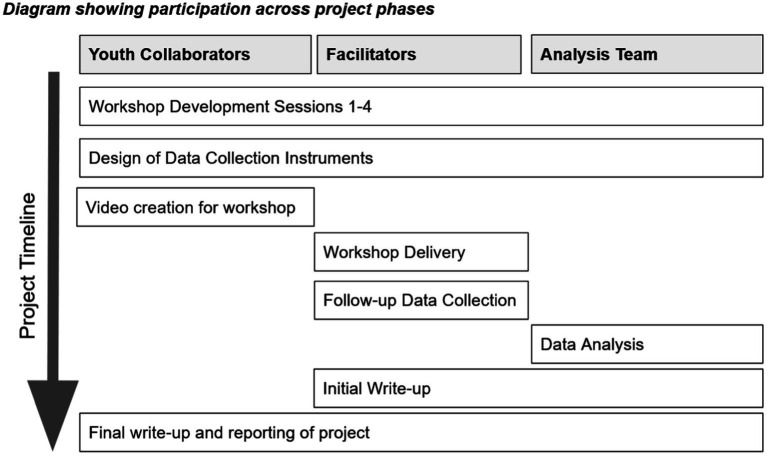
Co-creation diagram.

The research team consisted of three groups: Three youth collaborators (workshop co-creators), two facilitator-academics (workshop co-creators and facilitators – one clinical psychologist and one teacher) and four academic researchers (workshop co-creators and data analysis team). All researchers were involved in data interpretation and write-up. There were four phases: workshop development, workshop delivery in a school with sixth-form students, data analysis and write-up (Figure 1). The study received ethical approval from the University of Bath Psychology Ethics Committee (21–234).

### Phase 1: Co-creating the workshop

The three youth research collaborators were aged 19–25 (one male, two female) with lived experience of distressing eco-emotions, and were paid for their participation. Over 3 months, the research team met for four, 2-h meetings, using an iterative and collaborative process. Workshop design discussions were facilitated by the first author, following YPAR principles, with youth collaborators fully involved in the workshop design and decisions regarding key element for inclusion. The result was a 3-h structured workshop designed to support sixth-form students (i.e. those aged 16–18 years, in the final 2 years of secondary education) to express their beliefs and feelings about climate change and related issues, followed by reflection about what a hopeful future might be. The youth collaborators contributed to choices of research tools (recruitment, materials and post-workshop survey), illustrators, activities, technologies and techniques (pair work, individual work, group discussion, etc.). See Table 1 for workshop outline.

**Table 1:** Workshop outline.

Part 1 Opening and building the group. Engaging with the issues intellectually	Setting up and building the group as a community and “psychologically safe” space by agreeing clear ground rules including confidentiality and spending time in getting to know each other. Finding out from the group what they understand and find important about climate change and related issues
Part 2 Engaging with the issues emotionally	Exploring the emotional impact of climate change (eco-emotions) by watching a video of other young people talking about this, reflecting individually on personal responses and then sharing their experiences with one other person and then the whole group.
Part 3 Opening to the possibility of hope and a hopeful future	Exploring in general what hope means to each individual and how it feels, using a group-based activity. The facilitator leads a guided visualisation for all participants which explores their imagination of a hoped-for future, in the face of climate change and participants follow this with creating their story in whatever medium they prefer (writing, drawing, etc.).
Part 4 Moving forwards with realistic hope. Ending	Each individual shares their imagined story with the group, and this is followed by a group discussion. The group view the illustration created by the live illustrator and spend some time reflecting upon the whole workshop. The group ends with a brief gratitude exercise and debrief.

### Phase 2: Workshop delivery

The workshop was piloted with sixth-form students at their school. The school was identified through professional networks of the research team, as a state school local to the area in the South West of the UK. The workshop was delivered within a classroom on the school site in April 2022. It was delivered by two members of the research team with relevant experience (a clinical psychologist (E. Marks) and a teacher (LH)). A live illustrator was present throughout, translating the conversations and stories of the group into line drawings that aimed to capture core aspects of the discussion, and these pictures were shared both during and after the workshop. Participants were remunerated for their time and received refreshments. The study was advertised through the school, during assembly and *via* email, and participants signed up *via* an online form (Qualtrics). Those wishing to take part provided full, informed consent and a contact email, to which invitations were sent. Six students consented and four attended the workshop in April 2022. Table 2 shows the participant characteristics.

**Table 2:** Participant characteristics, including degree of current concern about climate change.

Name	Age (years)	Gender	Ethnicity	How worried about climate change (from 0 not worried to 4 extremely worried)
P1	17	Male	White British	Moderately worried (2)
P2	16	Male	White Other	Very worried (3)
P3	17	Female	White British	Very worried (3)
P4	18	Female	White British	Very worried (3)

COVID-19 meant that the illustrator attended remotely, sharing live the illustrations they had drawn, *via* a screen at the front of the classroom (see Supplementary material for images). The workshop was audio-recorded, transcribed and analysed. Post workshop, the participants received a copy of the illustrations and completed post-workshop surveys at 1 and 4 weeks, at which point they were asked to give anonymous feedback about the group, and any impact.

The workshop involved four parts (Table 1). Part one built a supportive group milieu, and facilitators emphasised the value of everyone’s contributions as they elicited participants’ understanding of climate change and related issues. Part two helped participants to explore their emotional responses to climate change, supported by sharing videos from the youth collaborators where they spoke about their own experiences of eco-emotions and distress, followed by pair work. After a break, part three helped individuals to contact a general sense of hope, followed by a guided visualisation of their hoped-for-future. Participants had time to then express their stories of hope in any form, and in part four, each participant shared their story. This was followed by a final reflective discussion about the workshop. The workshop ended with a grounding gratitude exercise and debrief.

### Phase 3: Analysis of outcomes

The workshop was analysed in three ways. Part 3a involved a thematic analysis of the workshop following Braun and Clarke (2006) methodology. Part 3b involved an emotion/sentiment analysis of the workshop transcript, to identify the most frequently occurring emotional responses and part 3c involved a content analysis of the post-workshop surveys. The methods and results of each of these are described in turn.

#### Analysis 3a: Thematic analysis of workshop transcript

The script of the recorded workshop was analysed using reflexive, inductive thematic analysis (Braun and Clarke, 2006), by two of the analysis team, independently of the workshop facilitators. Thematic analysis allowed for flexible application, pertinent to our findings not stemming from a particular theoretical position but being grounded in the data itself. The workshop audio-recording was transcribed using *otter.ai*, and the researchers familiarised themselves with the data by listening to the transcript to identify key moments, phrasings and semantic themes. Notes were taken and ideas marked for subsequent coding, which were confirmed or ruled out after listening to the recording again. After preliminary detection and tracing of key themes of analysis, these were cross-checked with a targeted process of manual transcription by a member of the research team. Inductive thematic analysis involved close reading of the transcript for coding based on semantic (i.e. linked to word choice), latent (more underlying in the point being made) or responses (in how the participant responds to a set question). This led to generation of initial codes, allowing for the further identification, illumination and organisation of key moments in the transcript where particular themes were apparent. These initial codes identified key features of certain sentences or phrases present in the transcript, that held semantic, latent or response value. Finally, themes were identified, reviewed and named by re-reading the transcript to see how when read together, themes that were shared across the transcript emerged. These emergent themes were reviewed and then coded across the transcript, allowing for the identification of how they might be clustered at certain points of the workshop and how they might relate to one another. Finally, these themes were given direct, describe labels. These different themes, overlapping and building on the initial codes provide the structure of the subsequent analysis section.

### Phase 3b. Sentiment and emotion analysis

A sentiment analysis of the transcript was performed in R version 4.1.3. This involved removing all text arising from speech by the facilitators or illustrator. These data were uploaded into R for preliminary data cleaning using the *tm* package. This involved transformations, including: removing special characters and extra whitespace from the text; converting all text to lowercase; removing common stop and filler words (such as “the” and “like”) and removing numbers and punctuation which here did not relate to assessing sentiment or emotions. The emotion and sentiment classification used the *syuzhet* package, which builds emotions on the NRC Word-Emotion Association Lexicon (EmoLex). This lists English words and their associations with eight basic emotions (anger, fear, anticipation, trust, surprise, sadness, joy and disgust) and two sentiments (negative and positive). These were classified in the NRC manually through a collaborative crowdsourcing process (Mohammad and Turney, 2013). To generate an overall sentiment score of the workshop session we conducted a sentiment analysis, which is a natural language processing technique used to determine word valence (positive, negative, neutral). Sentiment analysis is based on lexicons, here a selection of words with two polarities that can be used as a metric in sentiment analysis. There are many different types of lexicons that can be used depending on the context of the data. Sentiment analysis allows words to be represented on a numeric scale, to better express the degree of positive or negative strength of the sentiment contained in a body of text. Here the Syuzhet sentiment dictionary was used, with an extraction tool developed in the Natural Language Processing (NLP) group at Stanford.

### Phase 3c. Content analysis of survey (method)

Content analysis of all responses to the survey at 1 and 4 weeks was conducted by one researcher (JG) independently of the facilitators. This involved coding all the responses, by question and grouping these into themes (explicit and implicit) (Smith, 2000). Initial themes were first organised by question and following discussion with the research team, were then refined with codes and themes reorganised across both surveys together.

## Results

### Phase 3a: Thematic analysis of workshop

Four overarching themes were identified and are described below: (1) Multidimensional understanding of climate change; (2) Climate change engenders painful emotions; (3) Positives of climate change when it brings people together; (4) Hope for tomorrow lives today. These themed responses were defined by the authors iteratively, emerging from the analysis, not consciously selected, or defined by the participants. This series of themes represent the ways that participants ‘made sense’ of climate change, eco-anxiety and their individual and shared futures. They are neither linear nor in competition but represent component parts of the participants’ responses.

#### Theme 1: Multidimensional understanding of climate change

Even with a small group of four, there were various outlooks and beliefs about climate change, and related concerns. All saw climate change as a significant issue, beyond carbon emissions and global heating and relevant to many other environmental and social issues. All participants demonstrated a comprehensive understanding of climate change, its impacts and consequences. Melting ice caps, flooding and increasingly extreme weather were mentioned. This included recognising such uncertainties and impacts in day-to-day experience. One participant described how the weather *“is more unpredictable and more extreme…we had snow here last week, and then sun a few days before, [which] kind of just demonstrates that*” (P4). Participants went beyond assumptions of climate change as purely having meteorological consequences and discussed the other consequences of a changing climate including food insecurity, emergent vulnerabilities linked to health and disease and population displacement.

Furthermore, participants illuminated political and socio-economic elements of climate change. They talked about concepts of responsibility (what and who is responsible for historic and contemporary greenhouse gas emissions) and pervasive climate injustice (in geographic, economic and generational terms). In one discussion of the impacts of climate change, a participant was quick to highlight the relative privilege and resilience of those living in the UK to such problems: *“In the UK, we can afford importing [food], whereas other countries may struggle to do that*” (P1).

These discussions were not directly supported by evidence (scientific, anecdotal or otherwise) but still clearly demonstrated the multidimensional understandings of climate impacts amongst these young people who all recognised that climate change has multiple implications with socio-economic, political and cultural factors. When the facilitator asked participants what terminology they would use in describing such impacts, two participants sought more expansive terms:

“*Climate crisis* and *climate emergency* sort of give the sense of how dangerous it can actually be… *Climate change* seems like less of an impact [in the] long term?” (P1)

“[On the term *climate* emergency] … It’s quite a heavy word… We need to do things. It can seem a bit scary but it’s also important to us, so you can’t just gloss over it. *Climate change* – yeah, things change all of the time.” (P4)

Participants’ language and the emergent vocabulary of *climate emergency* perhaps address the ineffectiveness of previous terminologies and narratives of climate *change*, which failed to adequately communicate the danger and importance of the situation. ‘Emergency’ indicates how significant climate change and its impacts are to this group, and their broader cohort or generation (suggested by the use of the term ‘us’). This described significance was often presented by participants in negative terms of loss, frustration and anger in the second theme.

#### Theme 2: Climate change engenders painful emotions

When asked to reflect upon and discuss their individual emotional responses to climate change and climate action, participants expressed multiple emotions. The variety chimes with other research into eco-emotions, all of which suggest a heterogeneity in people’s responses (Hickman et al., 2021). Participants linked their emotional responses to specific thoughts, beliefs and experiences. Participants also recognised how these emotions affected their understanding of climate change, behaviours and visions of the future.

All participants described sadness and grief, related to a strong sense of loss, in particular was anticipatory grief about the future they felt unable to have. For example, one participant described grief as they felt unable to have children in the future, due to the changes and impacts that a changing climate might bring and restrict opportunities in the future:

“...mainly worrying but maybe also a sense of sadness of not being able to do things that I originally wanted to do… I know a lot of people who think about if they ever want to have children or not, based on the fact that the world in 40-years’ time may not be one which is as habitable as it is currently.” (P3)

This represents a sense of individualised consequences whilst recognising such losses will also apply to others in their generation; that the future lives they now anticipate will be different from the lives that they and many people in previous generations had hoped for and enjoyed up until now. Participant 3 described this as a mismatch between “*[the] sort of things I want in my life… and how they may look different than originally thought, due to the possible impacts of climate change.”* (P3).

There was a sense of uncertainty about what a ‘normal’ life and future might be. Sadness was sometimes replaced or joined by worry, stress, anger and frustration, emotions which were more often directed at others, such as those responsible for climate change. Participant 2 articulated anger towards those in business who have “*so much money, they have power, yet all they choose to do is exploit it for gain and profit, when they could be helping people or planet*” and who will not reduce emissions because “they are *just interested in growing their business*” (P2) and indicated that this caused him “*stress and worry*.”

These emotional responses were associated with how participants were making sense of their own responsibility for climate change or role in climate action. They recognised that though important, the overall impact is small if others do not act similarly, which could lead to difficult feelings such as guilt, anger and confusion:

“I agree about being responsible… I try and do all my things (but) I feel sort of angry, because it makes me feel stressed, that most of my clothes, are second-hand and I’m vegetarian, but then I feel guilty if I don't always follow that or like if I buy something new when I when in fact, my small impact of not buying something brand new is, although it's good, it's like the bigger companies and people with more power not doing anything. It makes me angry that I'm not, that I’m doing my best but then still has such a little impact. Also, that I feel… I should do that, because that’s what’s right. And then other people don’t… why is it that not everyone feels like this?” (P4)

This indicates a sense of responsibility which shapes behaviours aligned to one’s moral code, and which therefore feel like the right thing to do. However, the sheer scale of the problem complicates how helpful this feels to the individual, exacerbated by the uncaring behaviours of other people, including those in power, because these dwarf the impact an individual’s efforts have, which engenders frustration, and a struggle to understand how others might not care. This was not universal, however, and in a slight contrast, Participant 1, although ‘kind of worried’ reported becoming less worried when finding out about “new technology benefiting climate change in a good way”(P1).

Across the group, as the young people explored and defined their feelings about climate change, there was a strong desire to move to action, both individual and collective, to channel the more painful eco-emotions into something more positive, through finding a way to effect change. This was evident in how participants presented climate action using a different vocabulary, with it discussed in more-positive terms.

#### Theme 3: Positives of climate change when it brings people together

Despite their worries and the emotional burden, participants found some positives about the effects that the climate emergency is having upon society. Participants spoke about how climate action could bring people together and give them a sense of community. Participants often referred to the Fridays for Future movement (also commonly called the School’s Strike for Climate Movement) started by Greta Thunberg in 2018. This movement was present in the local city, with numerous protests staged by young people, and Greta Thunberg visiting in 2020. This rally was referred to by participants as a real benefit of the climate movement.

This created a sense of community which went some way to allaying the negative emotions detailed above. Participants reported that being part of climate action created a feeling of safety and made it feel “*that things will be ok*” (P4). This sense of things seeming brighter is, at times, rooted in feeling solidarity with the climate movement. Such solidarity not only stemmed from collective action but also from the process of *talking about how people feel* about climate change and its associated impacts, and having a space to share and hear these emotional impacts, rather than just thinking about what might happen next:

“A lot of the time, I guess that these conversations are more about what you think’s going to happen, rather than how people actually feel about it... I feel like having conversations with people can help you understand how it affects us, or not.” (P3)

These positive elements of climate action were often discussed by participants as giving rise to a sense of hope for change and hope for a better future. It is this that we turn to below.

#### Theme 4: Hope for tomorrow lives in today

The second half of the workshop was designed to allow participants space to express and present their stories of hope, and this emerged from the discussions. These stories were presented in numerous ways, including in speculative descriptions of what *might* be, drawings and poetry. When discussing how positive emotions might give rise to a sense of hope, participants offered a creative and imaginative vision of the way in which the future could be more hopeful. For some this involved the hope brought to them through technological advances. One participant spoke of virtual and augmented realities, cryptocurrencies and non-fungible tokens and how such emergent technologies could alter how we live, interact and consume goods and materials (P1). This represents a continuation of the cornucopian vision of climate futures, in which emergent technologies support climate change mitigation and adaptation.

However, other participants identified key opportunities for hope in everyday moments and interactions. This illuminated micro-level possibilities for hope—the small and the everyday, of life going on, of things both within and anthropocentric control. All of these were ground in opportunities for *changing* practices and dynamics, rather than possibilities and processes that are already available to us. Participant 2 highlighted the opportunities in being proactive and in more people, both individually and collectively, being ‘green’ in their behaviour. This includes the opening up of new wildlife reserves and the protection of landscapes and ‘*doing things like doing more reserves… for the preservation of animals for species, but also for the fact that, we can… still experience them”* which was seen as important as taking care of more vulnerable people by “*creating more opportunities for people to reduce things like homelessness, or poverty’*. Aligned to this, Participant 4 spoke about how their sense of hope is based on humanity’s ability to be kinder, although this may not always feel achievable to them.

“…people being more kind to each other… and acting in a more thoughtful way, not massive changes… but if everyone were just a bit nice, a bit more thoughtful, there would be big changes.” (P4)

A pertinent vision of hope, highlighted by other participants as effective in communicating a hopeful future, was found in how one participant presented a poem and the image involved of a flower breaking through concrete. We reproduce this poem below, with permission from the participant (P3):

Tall glass buildings and concrete streets,

As sky begins to melt into a golden pink

I saw that no one had noticed.

A city so vast, yet every inch is crammed with buildings

and where infrastructure lacks, concrete is plentiful.

No birdsong you will find here

Only the sound of people and mechanics as the days blend into one.

I saw it trying to emerge from beneath the pavement.

It had used all its strength to push through the stones,

In the hope, the desperate hope, that it would be seen.

A flower, the lonely flower

The first one I’d seen for real,

Only in photographs or descriptions had I encountered them before.

As I touched it the petals collapsed to the floor

But if there was one, I’m sure there can be more.

Participant 3 reflected that this might not be a particularly hopeful future, as highlighted by the sense of desperate hope and the statement that ‘*It’s more of a negative future, but there’s hope at the en*d’. The poem represents a reflection of how, even in a heavily anthropocentric, mechanised, urbanised and concreted-over world, nature might still find a way. It also represents the recognition that in the future we will face many challenges, so hope may need to be found by paying attention to, and caring about that which remains, even if the future itself will involve many losses and difficult changes. Notably, in this poem, other than their noise, the sense of people is absent from the scene. In contrast to other participants who saw hope in other people taking opportunities to be kind, proactive or to use new technologies, this poem signals how hope might also be found in the more-than-human. This image resonated strongly with participants, who highlighted the similarities between it and the 2008 film *Wall-E*, in which a robot finds new signs of life on a planet long-abandoned and over-polluted by humans; in spite of everything, a flower might still grow, and from this, we can ‘rebuild’ (P2).

### Phase 3b: Emotion and sentiment analysis of participant speech in workshop

**Figure 2:**
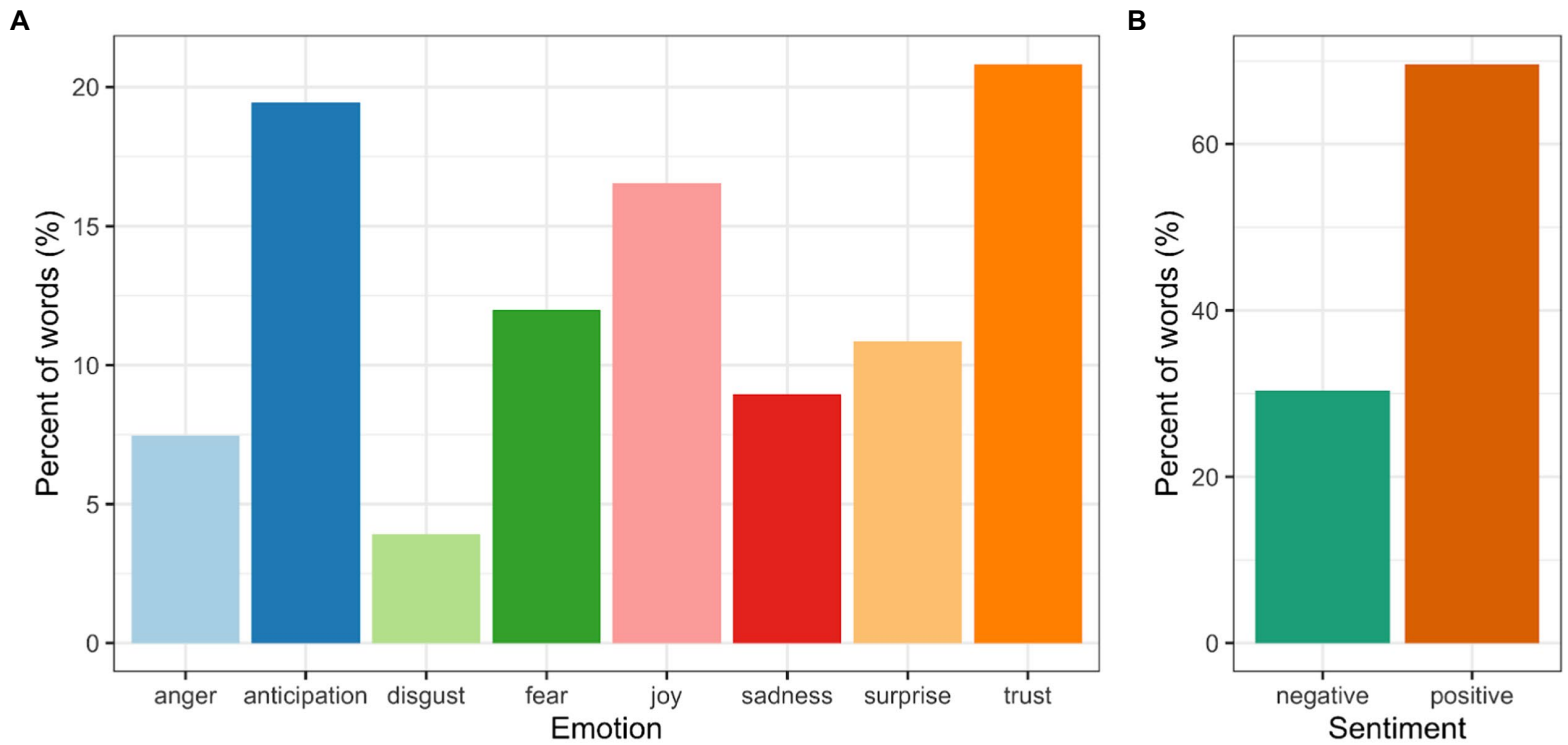
Sentiment analysis of workshop transcript. **(A)** Emotion classification of workshop transcript. **(B)** Sentiment classification of workshop transcript as percentage of meaningful words from the workshop transcript text.

The sentiment analysis included both positive and negative sentiments and a range of emotional expression. The three most common emotion groups were trust, anticipation and joy, with more negative emotions (fear, sadness, anger and disgust) occurring less frequently (See Figure 2).

### Phase 3c: Content analysis of survey

All participants completed the survey at 1 and 4 weeks. With only four participants, results were analysed for both surveys together, and they mirrored the positive experiences found in the sentiment analysis from the group. Three of four participants reported finding the workshop largely a beneficial experience (e.g. ‘great’, ‘enjoyable’ and ‘inspiring’), with one describing the experience as more mixed. Despite this, at the 1-month follow-up, all four participants stated that they would encourage others to attend the same workshop. There were three key themes that emerged regarding the workshop and the outcomes. These were:

1. Listening to others and hearing others’ thoughts and experiences

Most of the participants (¾) commented on how interesting and positive it was to listen to others and hear different perspectives. This aspect seemed to be particularly important for participants as it was mentioned repeatedly throughout the 1-week and 1-month surveys.

“I found it interesting and enjoyable as you got to see how other people's perspectives and views on climate change are different and vary between people.”

2. Expressing emotions and personal thoughts and feelings

As well as hearing from others, all participants commented on how much they valued having a space to express how they feel about climate change, and particularly that there were a variety of ways in which this was facilitated. Participants found the workshop safe, relaxed and non-hierarchical between students and facilitators, which allowed them to be more honest about their feelings, especially by allowing space for creativity:

“I also enjoyed being able to express myself creatively rather than just talking to express my emotions.”

They appreciated the creative nature of the expression, with positive feedback regarding the live illustration to summarise the discussion. The images themselves can be seen in the Supplementary material, with the first image summarising their understanding of the climate crises and the second image summarising ways of imagining the future from the perspective of realistic hope.

“I thought they were a nice way to capture the atmosphere and the conversation, without writing it all down. They are easier to look at than lots of words.”

After 1 month, participants were generally more aware of their feelings or the impacts of climate change. However, they were also reported being better able to manage such feelings. Although the workshop did not change how participants felt about climate change, with concern naturally remaining, this outcome was important to them. Even though they felt more aware of their feelings, they remained feeling better able to manage such feelings, even 1 month after the workshop.

“I think that since attending the workshop I feel more aware of the impacts of climate change, however I do not stress over it.”

3. Hope alone is not enough

One participant at both time points stated that they were not sure of the purpose of the workshop, and that more action is really what is required:

“Hearing people talk about hope didn't really make me feel more hopeful about climate change because hope won't change things, we need actual action. It seemed like we were trying to pretend like hope would make climate change better but it won't. I'm not really sure how hope will help.”

This indicates that for some participants, having a workshop focused on emotional expression and developing hope felt inadequate, and that future workshops might need to consider ways of bridging this gap between developing realistic hope and building towards meaningful action for climate change, an important learning point for future research in this area.

## Discussion

This novel pilot study, developed using principles of YPAR, explored how young people could share thoughts and feelings about climate change with their peers. It demonstrated that a facilitated space could help them to acknowledge the challenges and realities of climate change and offer a creative means to explore new stories of hope, grounded in reality. The young people attending this workshop clearly understood the climate crisis and expressed a mixture of painful eco-emotions alongside hope and more pleasant emotions. The results indicate that young people highly valued having a dedicated space to express their eco-emotions together. This appeared to enable them to make more sense of their thoughts and feelings, and they recognised that they are not alone in feeling this way, and that such emotions are a sign of how much they care about the world. They articulated their feelings and hopes for the future through the use of storytelling and creativity. Tracing these thoughts and feelings throughout their discussions allows for an understanding of the relative importance of talking about climate change, eco-emotions and hope in a group, the forms they arise in and how future work might build upon this. This process supports the claim that narrative approaches to eco-distress can offer unique and powerful ways to support young people in coming to terms with the reality of the crises, creating space where the difficult feelings can be experienced, without being paralysing, and as a result, creating opportunities for creative thinking to find new ways of thinking about the future.

The participants were both knowledgeable and concerned about climate change already. They demonstrated a comprehensive understanding of multiple issues from causes and immediate impacts to future consequences and the broader global context, including climate justice, the moral implications of climate change and how it affects those most marginalised and vulnerable, who have contributed the least to the problem. The language used by participants spoke to the importance of framing climate change in specific ways, and they claimed a vocabulary of ‘emergency’, ‘breakdown’ or ‘crisis’ when talking about these issues. Overall, these findings indicate that these young people were literate in issues of climate justice, and recognise how this is relevant to inter-generational factors and to the intra-generational and intersectional justices of today (Damico et al., 2020). Participants were fully aware of issues such as food poverty, international economic inequality and the links between climate change and human and planetary health, showing an appreciation for the interconnected nature of humans with each other, and the natural world. Respondents were able to describe a nuanced and mixed experience of the emotional responses to climate change, and how it shaped their thoughts and behaviours in relation to themselves, the world and other people. As the workshop progressed, what emerged was not a reduction or change in the painful experiences of climate change, but rather the emergence of a sense of realistic and engaged hope about the future that was held *alongside* the sadness, worry and anger. This sense of hope was necessarily linked to engagement with action at various levels, which aligns with existing research that shows the importance of *constructive* and *realistic* hope when engaging young people with climate change (Ojala, 2012).

As evident in both the workshop and the post-workshop surveys, the participants were keen to talk about both climate change *and* their emotional responses to it, an opportunity that is rarely available to young people in a school setting, or even in life more generally (Hickman et al., 2021). Providing this space for participants to discuss climate change and eco-emotions allowed participants to reflect upon, devise and present visions of how they understand, interact with and respond to climate change in emotional terms. Feedback clearly showed that this opportunity was warmly welcomed by participants, who benefitted from sharing their eco-emotions in a group setting with their peers and working in creative ways. This was evident from feedback after the workshop and discussions within it where they talked about the positive experiences of the broader youth climate movement, such as Fridays for Futures, and is consistent with other findings in the literature (Pihkala, 2020).

When participants were able to articulate their emotional responses to climate change, their reflections, vocabularies and emotions could be seen as ‘negative’, with experiences such as anger, frustration, sadness and loss. These emotions were inscribed with the multidimensional reading of climate change defined in the paragraph above, with participants recognising the moral implications as they described injustice in terms of different levels of vulnerabilities to and responsibility for climate change impacts. They recognised their own vulnerabilities here, which for this group was focused upon how climate change impacts will restrict the personal opportunities available to them, such as having their own children. Of note is that the focus was never exclusively just on climate change, and the young people talked a great deal about the multiple impacts and knock-on consequences on other people, and the natural world. Although the valence of these emotions can be construed as ‘negative,’ it is important to recognise how rational their discussions are, reflecting a sound understanding of the reality of what the world is facing today, and their ability to see themselves as part of an interconnected world, and show deep care, empathy and compassion for the pain and suffering of other humans and more-than-humans across the world.

Interestingly, despite the painful emotions aired in the group, the workshop also elicited many emotions and discussions with a positive valence. Emotion and sentiment analysis of participant speech in the workshop highlighted a greater level of positive sentiments, over negative ones, even though the workshop itself was split quite equally in terms of the exercises focused on climate change and eco-emotions and those focused on hope. Such positive sentiments included discussions of care, trust, gratitude, joy and hopeful anticipation as participants were able to think about the reasons they cared about these issues, their sense of gratitude and appreciation for many aspects of their lives and the anticipation of possible change when people work together.

When the workshop turned to specific discussions of hope, the conversations were wide-ranging, and it is notable that each participant described finding hope for a different future in very different ways, from emergent technologies to human kindness. The presentation of these hopes was as varied as the participants, and all involved an element of creativity, from poetry to visual art, and open-minded speculation. Participants articulated visions of alternative futures, in which we are kinder and more proactive, in which nature is protected and in which flowers can still grow through concrete. The adoption of creative means to present these visions appeared to support the sharing of new visions and stories of hope as previous authors have suggested (Pihkala, 2020).

A significant finding, particularly considering the small sample size, is that not all participants could find ‘hope’, or even see it as the desired outcome. As Participant 4 put it: “I *find it slightly hard to think of a completely hopeful version of the future. I think in a way… it does seem like we are not necessarily going in a good direction.*” This is such a crucial element when exploring these thoughts and feelings with young people, and what hope should mean or look like. After all, as reported by one participant in the feedback survey, ‘th*e emotion of hope can only get you so far’*, and what happens next is extremely important. Future research should carefully consider ways in which psychologists and educators can instil *constructive* hope. Evidence suggests approaches to support this should focus on connecting with like-minded communities engaged in inspirational action, to build a sense of shared care or to share stories of relatable ‘climate heroes’, emphasising that action can have an impact and that transformation is possible. Further psychological skills training to teach dialectical thinking might be helpful, so young people can learn to hold painful emotions and despair alongside constructive hope (rather than naive optimism). As a recent review of the literature demonstrates, simple optimistic messages about progress may not be of help to those concerned about the crises, and instead solution-oriented action (individual and collective) builds realistic hope and resilience (Ojala, 2022).

Creating visions of hope together, in relation to climate change may allow for participants a greater space to discuss, reflect upon and articulate their eco-emotions. However, hope should not be forced where it is not present, because the realities of climate change at times may feel hopeless, and this valid response must be honoured when it arises. Furthermore, the feeling of hope is not ‘enough’, and this type of approach should not be limited to helping people ‘feel better’. Rather, participants recognised that the usefulness of a workshop like this is that it can lead to building community with others and further actions, which importantly includes starting to talk more to other people about these issues. Stories of hope are more than imaginings; they must be understood, shared and form the ground upon which further climate action can be built. These stories offer new visions of socio-economic and political changes from which new theories could be built (Bowman, 2019). However, whilst workshops like these are important for exploring emotions around climate change, it is still no replacement for climate action from big businesses and government; in other words, prevention is better than cure.

### Strengths and limitations

This study is one of the first of its kind to report on the ways in which young people can talk together in a peer-group setting to explore eco-emotions and potentially find a way of feeling less isolated and distressed by these. The approach included a mixture of intellectual and emotional engagement with the issues, building a group community to share experiences, and using imaginative means to visualise more hopeful futures. A significant strength is the use of the YPAR approach, with the research design co-led by young people. The appreciation of the participants and likelihood they would recommend this to other young people indicated the final workshop was both acceptable and valued. The writing of this report also involved young people, so youth perspectives are represented throughout the entirety of the research process. The main limitation of this study is the small sample size, which reflects the pilot nature of the group. There are also limitations regarding the representative nature of the sample. All participants were white, and the lack of diversity limits the generalisability of the results. All had opted to take part in the workshop, which might have led to a selection bias of people already engaged and potentially more knowledgeable about the climate crisis than their peers. They also had high levels of eco-distress, which may not reflect the average level of concern across their peers, and so the results may mainly reflect the views only of young people who are worried about climate change. The findings are therefore tentative, and clearly repeated workshops and larger-scale groups are required for us to deepen our understanding of the processes and outcomes of this type of workshop across. The main finding of this project is that the methods used here are likely to be acceptable and valued by young people, and that this type of approach may offer a new way of supporting emotional expression for young people, who are increasingly distressed by climate change, but who have may rarely have a place to share these feelings.

## Conclusion

This study indicates that a facilitated, school-based workshop exploring eco-emotions and stories of hope can offer young people a rare and valuable space to explore and discuss their feelings and thoughts about climate change. In line with existing research, the young people here reported very painful feelings about climate change and recognised this as being a global crisis linked to other global problems including ecological destruction, social injustice, an economic model focused on wealth creation for the few and the inaction of those in power to act urgently to mitigate and adapt in a way that is fair across the world. These young people recognised the climate crisis as having a moral element that many people are failing to act upon, despite the ethical behaviour of some individuals, and this can make them feel powerless. They demonstrated emotional maturity in their ability to hold both painful eco-emotions whilst also looking for ways to feel hopeful about the future, and there was a strong theme relating to the importance of collective engagement as an approach that both supports individual distress and effective climate action. Hope is but one part of this. It is an imaginative force and nourishing emotion but as “hope will not help on its own,” it needs to be active and backed up by structural changes enforced by those in authority. With related action, these visions of the future can become more than wishful thinking, they help us all move together towards a future reality we can create together, for our global community and the generations that follow us.

## Data availability statement

The raw data supporting the conclusions of this article will be made available by the authors, without undue reservation.

## Ethics statement

The studies involving human participants were reviewed and approved by the University of Bath PREC 21–234. Written informed consent from the participants’ legal guardian/next of kin was not required to participate in this study in accordance with the national legislation and the institutional requirements.

## Author contributions

EMar led the manuscript. EMar, EA, JG, JA, and DS designed the study. EMar and LH delivered the workshop. EA and DS conducted the thematic analysis. JG analysed the content. JA analysed the sentiment. All authors co-designed the workshop, and contributed to and approved the final manuscript.

## Funding

Funding was provided by the GW4 Crucible Seed Funding, which made this work possible.

## Conflict of interest

The authors declare that the research was conducted in the absence of any commercial or financial relationships that could be construed as a potential conflict of interest.

## Publisher’s note

All claims expressed in this article are solely those of the authors and do not necessarily represent those of their affiliated organizations, or those of the publisher, the editors and the reviewers. Any product that may be evaluated in this article, or claim that may be made by its manufacturer, is not guaranteed or endorsed by the publisher.
