# Supplementary figures and images for: Stories of hope created together: A pilot, school-based workshop for sharing eco-emotions and creating an actively hopeful vision of the future
Source: Front Psychol. 2023 Jan 6;13:1076322. doi: 10.3389/fpsyg.2022.1076322 (PMC9853550; doi:10.3389/fpsyg.2022.1076322)

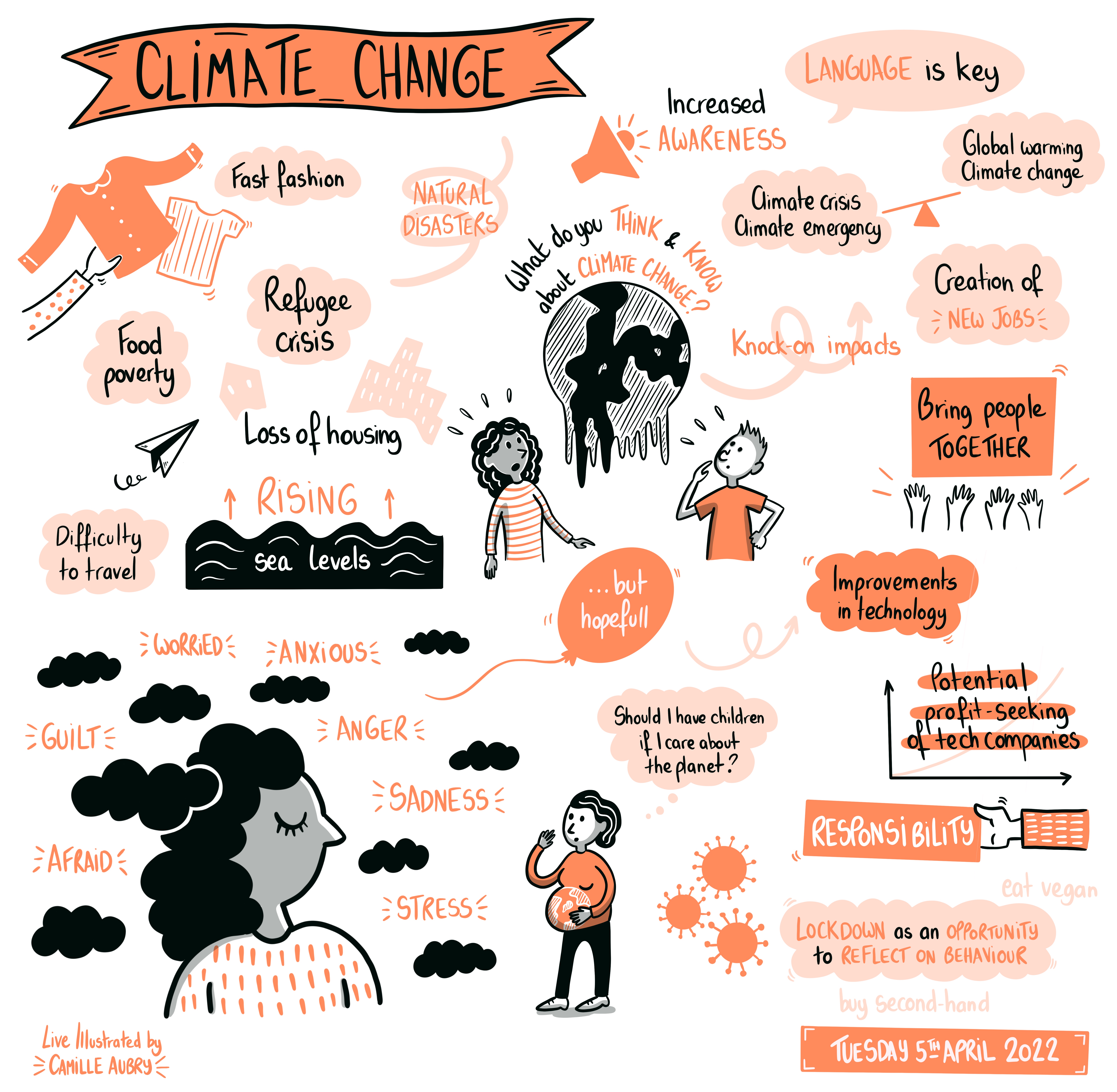

Supplement: Supplementary file 1 [file Image_1.jpeg]

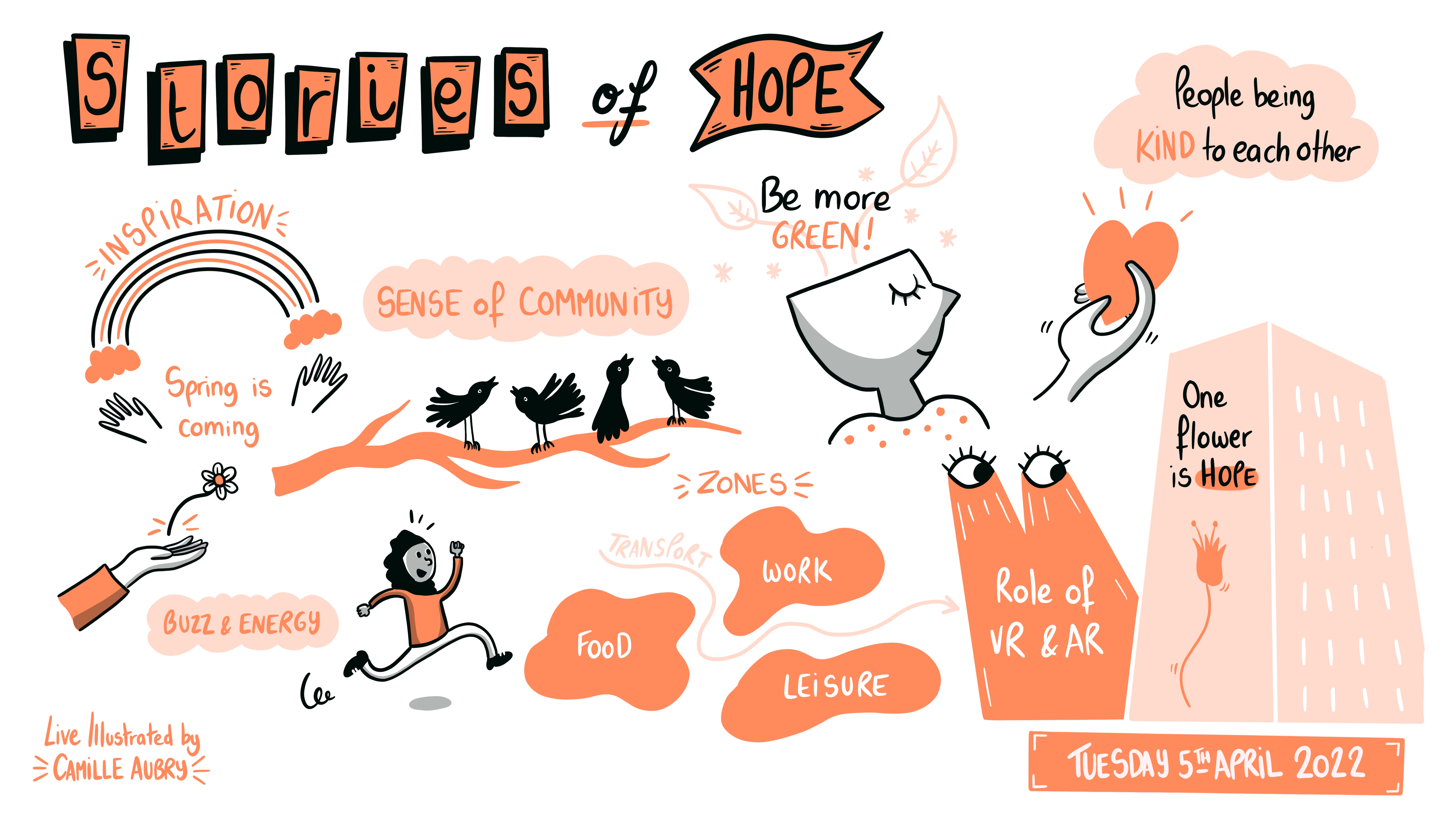

Supplement: Supplementary file 2 [file Image_2.jpeg]
